# The socioecological model levels, behavior change mechanisms, and behavior change techniques to improve accelerometer-measured physical activity among Hispanic women: a systematic review

**DOI:** 10.1186/s12966-025-01783-y

**Published:** 2025-06-19

**Authors:** Elizabeth Lorenzo, Jeni Page, Rebeca Wong, Elizabeth Lyons

**Affiliations:** 1 School of Nursing, University of Texas Medical Branch, Galveston, TX USA; 2 School of Nursing, Texas Tech University Health Sciences Center, Lubbock, TX USA; 3 Department of Population Health Sciences, Long School of Medicine, University of Texas Health Sciences San Antonio, San Antonio, TX USA; 4 School of Health Professions, University of Texas Medical Branch, Galveston, TX USA

**Keywords:** Exercise, Minority women, Behavior change, Socioecological factors, Health promotion, Disease prevention

## Abstract

**Background:**

Interventions to increase and maintain Hispanic women’s moderate-to-vigorous physical activity (MVPA) are lacking. Socioecological models hypothesize that MVPA participation is influenced by factors at multiple levels of the environment (i.e., intrapersonal, interpersonal, organizational, neighborhood, policy). These factors, including behavior change mechanisms (BCM), are targeted with behavior change techniques (BCT) delivered through interventions to improve MVPA participation. However, the specific factors and BCT that positively influence Hispanic women’s MVPA remain unknown.

**Purpose:**

Synthesize existing literature to determine the socioecological model levels, BCM, and BCT that significantly increased Hispanic women’s accelerometer-measured MVPA.

**Methods:**

Databases (PubMed, CINAHL, Scopus, Cochrane Library and PsychINFO) were searched using key terms ((Hispanic women) OR (Hispanic OR Mexican American OR Latina OR Latinx) AND (female) AND (exercise OR physical activity)) without date or geographic limitations in March 2023. Peer-reviewed studies published in English that tested interventions to increase Hispanic women’s accelerometer-measured MVPA were included. Two authors extracted data, a narrative synthesis was conducted, and a conceptual model of a multilevel MVPA intervention was proposed.

**Results:**

Nine unique interventions were identified with four studies providing additional findings (*N* = 13). Studies were conducted in the United States and totaled 2,303 Hispanic women (*M* = 28.4–44.6 years). Self-efficacy (intrapersonal level) and family participation (interpersonal level) were the only BCM that mediated MVPA post-intervention. Half of the studies targeting the organizational (*n* = 3/6) and 83.3% (*n* = 5/6) targeting the neighborhood levels demonstrated significant improvements in MVPA. BCT targeting confidence, goals, and problem-solving at the intrapersonal level, partner support and childcare barriers at the interpersonal level, and physical activity access with methods to overcome weather and safety barriers at the neighborhood level significantly improved MVPA post-intervention. None of the interventions tested for longer-term (i.e., > 9-months post-intervention) MVPA change.

**Conclusions:**

Interventions to increase Hispanic women’s accelerometer-measured MVPA in the shorter-term should be developed to include BCT to increase self-efficacy (intrapersonal level) and family participation (interpersonal level) and identify physical activity opportunities/access (neighborhood level), including BCT to overcome safety and weather barriers. Future research is needed to confirm these findings and determine the socioecological levels, BCM, and BCT to maintain Hispanic women’s MVPA in the longer term.

**Trial Registration:**

The 2020 Preferred Reporting Items for Systematic Reviews and Meta-Analyses (PRISMA) checklist was followed for this systematic review. The protocol for this systematic review was registered with the International Prospective Register of Systematic Reviews (PROSPERO), registration number CRD42021285063.

**Supplementary Information:**

The online version contains supplementary material available at 10.1186/s12966-025-01783-y.

Only half of adult women living in the United States met the national aerobic recommendations of 150 min per week of moderate-to-vigorous physical activity (MVPA) in 2018 [1], with only 43.8% of Hispanic women meeting recommendations compared to 54.7% of non-Hispanic White women [2]. Regular participation in MVPA prevents and improves cardiovascular health across the lifespan [1, 3] and any amount improves health in otherwise inactive individuals [1]. The significance of MVPA in improving cardiovascular health cannot be understated, as cardiovascular disease is the leading cause of death among all women [4] and second among Hispanic women in the United States [5]. This highlights the urgent need for effective interventions to increase MVPA and improve cardiovascular health among Hispanic women.

Research focused on the development and testing of physical activity interventions has been conducted for decades [6]; however, low levels of MVPA persist, regardless of age, sex, or ethnic or racial group [2]. One potential explanation for low MVPA participation among Hispanic women is that effective interventions are rarely designed to translate into context-dependent, real-world settings appropriate to the population of interest [7]. Socioecological models hypothesize that factors at the individual (i.e., intrapersonal) and extra-individual (i.e., interpersonal, organizational, neighborhood, policy) levels influence physical activity participation. These factors at different levels of the socioecological model serve as barriers to and supporters of positive behavior change making it necessary to target factors at multiple levels when developing and testing interventions to increase MVPA [8–10]. For example, access to safe, low cost physical activity resources has been identified as critical facilitators for Hispanic women to be physically active [11]. On the contrary, individual perceptions, lack of time related to caregiving and familial responsibilities, and cold weather [12] have been found to be barriers to physical activity among Hispanic women.

Behavioral interventions should also be developed to engage, measure, and test behavior change mechanisms (BCM), the malleable, critical mechanisms responsible for behavior change, to effectively improve the desired behavior [7, 13]. Self-regulation, stress resilience and reactivity, and interpersonal and social processes have been suggested as the overarching classes of BCM to target through behavioral interventions [13]. Additional evidence suggests self-regulation, motivational readiness/stage of change for physical activity, and behavioral processes of change as the BCM responsible for physical activity improvements among adults [14]. Interestingly, there have been no BCM identified that explain increased physical activity for ethnically diverse populations, including Hispanic women [11, 15–18]. The lack of knowledge and consensus surrounding the specific BCM necessary to improve physical activity among Hispanic women makes it difficult to develop effective MVPA interventions.

Behavior change techniques (BCT) are the observable and replicable strategies (i.e., active ingredients) delivered by an intervention to promote change in the specific BCM to mediate improvements in behavioral outcomes [19]. The content of BCT has been identified as a primary factor driving behavior change [20], including physical activity improvements [21–23]. It has been suggested that framing BCT within the context of the BCM may promote further refinement of BCT content and development of the physical activity behavior change processes [24]. However, details explaining the linkages between the BCT implemented and BCM targeted have been limited [25, 26], including in the physical activity literature [11, 15–18, 24, 27]. Furthermore, the specific combinations of BCT needed to increase physical activity has yet to be determined, warranting further investigation [21–23]. Understanding the BCT that facilitate change in the specific BCM mediating physical activity is necessary to streamline interventions to include only the most important, active ingredients [7, 13].

Most physical activity interventions implemented and tested among adults have targeted BCM using BCT at the intrapersonal and interpersonal levels [14] with limited BCT implemented at the organizational, neighborhood/environmental, and policy levels [14, 21, 22, 28]. Research exploring the linkages between the BCM and BCT in the context of the socioecological levels targeted related to intervention efficacy to increase physical activity for adults [14, 21, 22, 28] and ethnically diverse populations, including Hispanic women [11, 15–18], is lacking. In addition, subjective measures of physical activity were the primary outcome measure in previous reviews evaluating the efficacy of interventions to increase MVPA among samples including Hispanic women [11, 16–18]. Of these four reviews, only one disaggregated their findings based on physical activity measure, showing that 70% (7 of 10) of studies using subjective measures found significant improvements in physical activity compared to 60% (3 of 5) using device-measured physical activity for Hispanic and African American women [16]. The use of subjective measures potentially introduces bias [29] and MVPA has been overestimated by Hispanic women with self-reported measures in previous research [30, 31] necessitating the inclusion of accelerometer-measured MVPA to evaluate interventions.

Despite the importance of designing contextually appropriate MVPA interventions to maximize the implementability, efficacy, and sustainability of interventions [7–9, 32], investigations determining the socioecological levels, BCM, and BCT necessary to include in an intervention to increase accelerometer-measured MVPA among Hispanic women are lacking. The purpose of this systematic review was to: 1) determine if interventions increased and sustained accelerometer-measured MVPA among study samples of 100% Hispanic women; 2) identify which socioecological levels targeted increased accelerometer-measured MVPA; 3) identify which BCM mediated accelerometer-measured MVPA improvements within the context of socioecological levels; 4) identify the BCT within the context of the BCM and socioecological levels that led to an increase in accelerometer-measured MVPA; 5) propose a conceptual model of a multilevel intervention to increase Hispanic women’s MVPA; and 6) explore which health-related variables were included as secondary outcomes.

## Methods

### Eligibility criteria

Studies were included in this review if they met the following eligibility criteria: 1) published in a peer-reviewed journal; 2) written in English; 3) included apparently healthy Hispanic women; 4) an experimental design; 5) tested an intervention to increase physical activity; and 6) included the outcome of accelerometer-measured moderate and/or moderate-to-vigorous physical activity. Studies including pregnant women or a diagnosed chronic health condition as inclusion criteria were excluded. Studies with samples that included men or other races and ethnicities of women were eligible to be included if they disaggregated data to analyze the findings for Hispanic women separately. Conference abstracts and gray literature were also excluded. We did not impose a publication date limitation because this review had not been previously conducted.

### Search strategy

The literature searches were conducted January/February 2022 and updated March 2023 using PubMed, CINAHL, Scopus, Cochrane Library and PsychINFO databases. Supplementary File 1 includes the search strategy for each database with results. Searches were independently conducted by a medical librarian and citations were uploaded to EndNote and then imported into Covidence software. Duplicates were removed and two authors (EL, JP) individually reviewed study titles and abstracts for inclusion based on the PICO (population – apparently healthy Hispanic women ≥ 18 years, intervention – physical activity/exercise, comparator – any or none, outcome – accelerometer-measured moderate and/or moderate to vigorous physical activity). Both authors reviewed the final list of titles and resolved any discrepancies, with a third author (EJL) consulted when consensus could not be reached. The final list of studies for inclusion were independently reviewed by two authors (EL, JP) in their entirety to verify they met eligibility criteria. The reference lists from all studies that met eligibility criteria and related systematic reviews that were tagged during the screening process were reviewed to identify additional studies for inclusion.

### Data collection

Two authors (EL, JP) independently extracted data into Covidence software from each study, including: 1) study setting; 2) sample characteristics; 3) study design; 4) intervention details (format, dose, frequency, and language); 5) BCM targeted; 6) measure of BCM targeted; 7) BCT used to engage BCM; 8) socioecological level targeted; 9) BCT or intervention component used to target each socioecological level; 10) specific accelerometer used to measure physical activity; 11) statistical analyses methods to test for change in BCM; 12) findings of BCM analyses and/or mediation analysis; 13) findings for accelerometer-measured MVPA outcomes (i.e., mean differences and *p*-value or statistical test value and *p*-value, when mean difference was not available); 14) follow-up findings; and 15) health-related outcomes tested for change. Operationalization of the socioecological model levels targeted through the interventions included: 1) intrapersonal – any intervention component targeting only the individual; 2) interpersonal – intervention components targeting social support/interactions; 3) organizational – intervention components that took place at community-based organizations (e.g., group physical activity sessions or group discussions at churches, malls, community centers, etc.); 4) neighborhood/environmental – intervention components targeting the neighborhood/community surrounding an individual’s home and/or the environment (e.g., maps of walking routes in neighborhoods, neighborhood safety, built environment, weather), including ways to overcome issues at this level; 5) policy – intervention components targeting policy change or advocacy related to physical activity. Data extractions were reviewed, discussed, and discrepancies resolved. When consensus could not be reached, a third reviewer (EJL) was consulted.

### Risk of bias assessment and grading of recommendations assessment, development, and evaluation

Version 2 of the Cochrane Risk-of-Bias tool for randomized trials (RoB 2), the Revised Cochrane Risk-of-Bias tool for cluster-randomized trials (RoB 2 CRT) [33, 34], and the Risk Of Bias In Non-Randomized Studies of Interventions (ROBINS-I) [35] were used to measure the risk of bias for the included studies. The RoB 2 domains include bias 1) arising from the randomization process; 2) due to deviations from intended interventions; 3) due to missing outcome data; 4) in measurement of the outcome; and 5) in selection of the reported result. The RoB 2 CRT also includes domain 1b, risk of bias arising from the timing of identification or recruitment of participants in a cluster-randomized trial. ROBINS-I domains include bias 1) due to confounding; 2) in selection of participants into the study; 3) in classification of interventions; 4) due to deviations from intended interventions; 5) due to missing data; 6) in measurement of outcomes; and 7) in selection of the reported result. Two authors (EL, JP) independently extracted data, compared findings, and discussed all discrepancies. A third author (EJL) was consulted when consensus could not be reached. The Grading of Recommendations Assessment, Development, and Evaluation (GRADE) tool was used to assess the certainty of the synthesized evidence of interventions to increase MVPA [36, 37]. The GRADE checklist included questions related to each domain, including methodological limitations of the studies, indirectness, imprecision, inconsistency, and publication bias. Applicable questions were answered and each GRADE domain was narratively summarized and rated as no concern (does not appear to be an issue), serious concern (some exists), or very serious concern (severe or from several sources) [36]. Borderline was added if the judgement for a concern was not clear cut and more on a continuum [37]. The certainty of overall evidence was determined starting with high quality and was downgraded by one level for serious concerns for each domain [36]. A statement to communicate the GRADE level of certainty was described [36, 38].

### Narrative synthesis

Study and intervention characteristics and findings (including proportions of significant versus not significant) were compared and synthesized, including: 1) study settings; 2) sample characteristics (age, Hispanic subgroup, income); 3) study designs; 4) intervention characteristics (i.e., language, delivery format, duration); 5) BCM targeted; 6) BCT for engaging BCM; 7) BCM testing; 8) BCM findings; 9) mediation analyses findings; 10) MVPA findings; 11) follow-up findings; 12) socioecological levels targeted; 13) BCT targeting socioecological levels; and 14) health outcomes tested. Follow-up findings included preliminary, and shorter- and longer-term, with shorter-term defined as ≤ 3 months and longer-term as > 3 months. These were not based on established cut-points but relative within the group of studies included in this review.

Findings across studies were narratively synthesized differently for socioecological levels, BCM, and BCT. Socioecological levels were synthesized and identified as effective for increasing accelerometer-measured MVPA if more than 50% of studies targeting the level demonstrated significant MVPA improvements at any time point after the primary intervention ended. BCM were only included as effective if they were found to mediate MVPA improvements. BCT at the intrapersonal and interpersonal levels were included only for the BCM that were found to mediate MVPA improvements. Of note, no BCM were explicitly identified at the organizational, neighborhood/environmental, and policy levels nor were mediation analyses conducted at these levels. Therefore, the BCT targeting the organizational, neighborhood/environmental, and policy levels were only included from interventions that significantly increased MVPA in more than 50% of the studies that targeted the specific level. The BCT from levels which increased MVPA in more than 50% of studies were then synthesized into themes across each specific socioecological level. A conceptual model of a multilevel MVPA intervention was developed based on the findings of the narrative synthesis.

## Results

### Study selection

**Fig. 1:**
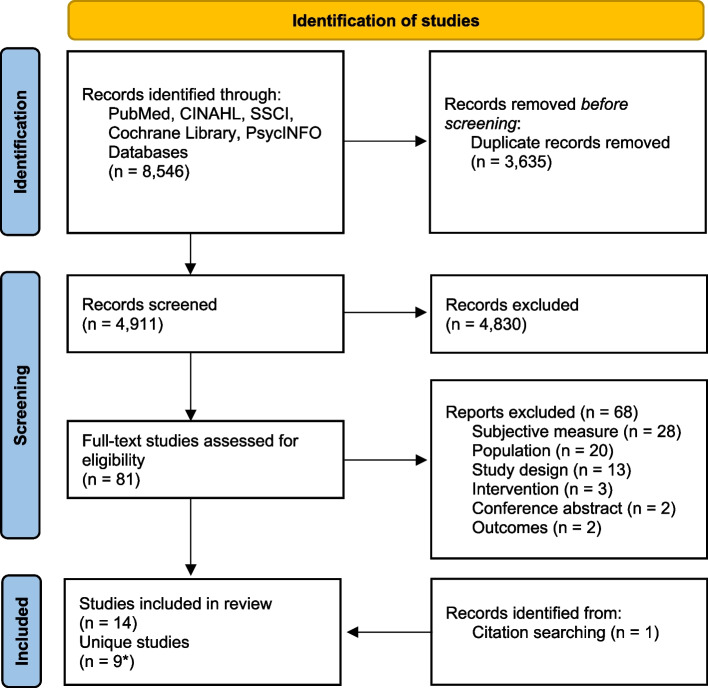
Diagram of PRISMA Flowchart for study selection. *Note*. *Nine studies are unique, five studies provide additional findings. Adapted from: Page MJ, McKenzie JE, Bossuyt PM, Boutron I, Hoffmann TC, Mulrow CD, et al. The PRISMA 2020 statement: An updated guideline for reporting systematic reviews. BMJ 2021;372:n71. https://doi.org/10.1136/bmj.n71

A combined total of 8,546 studies were identified. After duplicates were removed, 4,911 titles/abstracts were screened with 81 full-text studies reviewed for eligibility. A total of 14 studies were identified that met inclusion and exclusion criteria and are included in this review [39–52]. However, only nine [40–44, 46, 49, 51, 52] of the 14 studies were unique and the remaining five reported additional findings (i.e., preliminary, shorter-term, longer-term, mediation analysis) [39, 45, 47, 48, 50]. The nine primary outcome citations were used throughout the results section, except when reporting data collection time points and the additional findings, to allow for easier interpretation and prevent confusion of interventions that had more than one study that reported findings. See Fig. 1 for the PRISMA diagram.

### Demographic characteristics

Table 1 provides characteristics for each study sample. Studies were published between 2013 [44] and 2022 [40, 49] and all were conducted in the United States. A majority of studies were conducted in California [40, 43, 46, 49, 51] and one each in Rhode Island [44], Alabama [41], Arizona [42], and Texas [52] (see Supplementary File 2 for study settings). The majority of studies (88.9%) were randomized trials [40, 42–44, 46, 49, 51, 52], two of which were cluster randomized controlled trials [40, 52], with one quasi-experimental pilot study [41]. Six of the unique studies compared an intervention group (IG) with a control group ([CG] [40, 42–44, 46, 52]. CG consisted of cancer screening [40], non-physical activity health information [42], safety/disaster preparedness education [43], wellness [44, 46], and community health and safety [52]. One study compared two IG, the original *Seamos Saludables* intervention study [44] compared to a theory- and technology-enhanced version of the *Seamos Saludables* intervention called the *Seamos Activas II* [49]. One study had three intervention arms, of which the only difference was the type of step goal implemented, which included 10,000 steps per day versus 3,000 steps in 30 min versus a self-selected step goal [51]. Only one of the included studies did not include a CG or comparator [41].

**Table 1:** Evidence Table

Authors (Year)	Sample Characteristics	BCM Targeted	BCM Measure	PA Outcome Intervention/ Comparator	BCM Findings	Mediation Analysis	MVPA Findings
Arredondo (2017) [39] Arredondo (2022) [40]	*N* = 436 Mean age: 44.4 ± 9.6 yrs Income: < $24,000/yr −58.3% (*n* = 236)	Behavioral strategies for PA	Adapted PA and Dietary Management Questionnaire	MVPA IG vs CG (cancer screening)	Significant improvement in behavioral strategies for PA for IG vs. CG at 12-months, adj. mean difference 1.08, effect size = 0.42, *p* < .001	N/A	Significantly higher MVPA for IG vs. CG at 12-months, adj. mean difference 0.15, effect size = 0.25, *p* = .03 (preliminary findings) No significant differences in MVPA change for IG vs. CG at 24-months
Cherrington (2015) [41]	*N* = 35 Mean age: 37.7 ± 8.3 yrs Income: Not provided	Autonomy Perceived Competence Relatedness to Others	N/A	MVPA IG only Weight loss intervention	N/A	N/A	No significant changes in MVPA at 8-weeks Significant increase in MVPA from baseline to 6-months, unadj. mean difference 27.4, *p* < *.02* *Inconsistent reporting, table* p* = 0.291, *p* < .02 in text
Keller (2014) [42]	*N* = 139 Mean age: 28.3 ± 5.6 yrs Income: ≤ $20,000/yr—69.3% (*n* = 96)	Social Support for Exercise (SSE) General Social Support: -Emotional/ Informational -Tangible -Affectionate -Positive Social Interactions -Overall	Adapted Social Support for Exercise Survey Medical Outcomes Study: Social Support Scale	Moderate walking activity Moderate lifestyle activity Vigorous activity IG vs CG (non-PA health information)	Significantly higher SSE for the IG vs. CG at 6-months, unadj. mean difference 0.24, *p* = .028 Significant decrease in SSE for the IG vs. CG from 6 to 12-months, unadj. mean difference −0.31, *p* = .001 Significantly higher emotional/ informational support for CG (3.95, 3.95, 4.09) vs. IG (3.61, 3.19, 3.29) overall, *p* = .037 Significantly higher emotional/ informational support for CG vs. IG at 12-months, unadj. mean difference 0.40, *p* = .040 Significantly higher tangible support for CG vs. IG at 6-months, unadj. mean difference 0.53, *p* = .028 No significant group x time effects for general social support overall No significant change in emotional/ informational support at 6-months, tangible support overall or at 12-months, or affectionate and positive social interactions overall or at 6- or 12-months	N/A	Significantly higher levels of moderate intensity walking for IG vs. CG at 6-months, unadj. mean difference 13.6, *p* = .0008, and at 12-months, unadj. mean difference 15.4, *p* = .0207 Significant increase in moderate intensity walking for IG vs. CG from baseline to 6-months, unadj. mean difference 11.2, *p* = .007 No significant group differences in change in moderate intensity walking from baseline to 12-months or 6 to 12-months Significant changes in moderate lifestyle PA for IG (77.3, 102.2, 124.6) and CG (77.3, 90.6, 104.8) across time points but not between groups No significant findings for vigorous intensity PA
Koniak- Griffin (2015) [43]	*N* = 223 Mean age: 44.6 ± 7.9 yrs Income: ≤ $20,000/yr—54.7% (*n* = 122)	Goal-setting Self-monitoring	N/A	Moderate PA IG (lifestyle intervention) vs CG (safety/ disaster preparedness)	N/A	N/A	No significant change in moderate PA for IG vs. CG at 9-months
Marcus (2013) [44] Marquez (2016) [45]	*N* = 266 Mean age: 40.7 ± 10.0 yrs Income: < $20,000/yr—54.0% (*n* = 144)	Self-efficacy for PA The Processes of Change: -Cognitive processes -Behavioral processes Social support for exercise: -Family participation **-**Family rewards & punishment **-**Friend participation	Self-Efficacy for Exercise Processes of PA Behavior Change Social Support for Exercise Survey	MVPA IG vs CG (wellness)	Significantly higher self-efficacy for IG vs. CG at 3-months, adj. mean difference 0.52, *p* < 0.01 Significantly higher cognitive processes for IG vs. CG at 3-months, adj. mean difference 0.37, *p* < 0.01 Significantly higher behavioral processes for IG vs. CG at 3-months, adj. mean difference 0.66, *p* < 0.01 Significantly greater increase in family participation for IG vs. CG at 6-months (*t* = 4.07, *p* < 0.01) and 12-months (*t* = 2.32, *p* = 0.02) Significantly greater increase in family rewards and punishment for IG vs. CG at 6-months (*t* = 3.92, *p* < 0.01) and 12-months (*t* = 3.70, *p* < 0.01) Significantly greater increase in friend participation for IG vs. CG at 6-months (*t* = 2.16, *p* = .003) and 12-months (*t* = 2.61, *p* = 0.01)	Family participation mediated intervention effects on MVPA at 6-months, *ab* = 5.21, 95% CI: 0.91–14.11 No significant indirect effect of family rewards & punishments or friend participation on MVPA at 6-months or 12-months No significant indirect effects of family participation on MVPA at 12-months	Significantly more MVPA for IG vs. CG at 6-months, adj. mean difference 35.6, *p* < 0.01 Significantly more MVPA for IG vs. CG at 12-months, unadj. mean difference 31.2, *p* < 0.01
Marcus (2016) [46] Hartman (2017) [47] Larsen (2021) [48]	*N* = 205 Mean age: 39.2 ± 10.5 yrs Income: < $30,000/yr—66.4% (*n* = 136)	Self-efficacy for PA Processes of Change: -Behavioral processes **-**Cognitive processes PA enjoyment Social Support for Exercise: -Family participation -Family rewards & punishment -Friend participation	Self-Efficacy for Exercise Process of Change PA Enjoyment Scale Social Support for Exercise Scale	MVPA IG vs CG (wellness)	Significantly greater increase in self-efficacy for IG vs. CG at 6-months, adj. mean difference 0.29, *p* < .001 Significantly greater increase in behavioral processes for IG vs. CG at 6-months, adj. mean difference 0.67, *p* < .001 Significantly greater increase in cognitive processes for IG vs. CG at 6-months, adj. mean difference 0.59, *p* < .001 No significant findings for PA enjoyment, family participation, family rewards and punishments, or friend participation for IG or CG at 6-months	Self-efficacy mediated intervention effects on MVPA at 6-months, *ab* = 12.15, 95% CI: 11.25–16.34 No significant indirect effects for behavioral and cognitive processes of change, or PA enjoyment on MVPA at 6-months	Significantly higher MVPA for IG vs. CG at 6-months, mean difference 31.0, *p* < .01 Significantly higher MVPA for IG vs. CG at 12-months, mean difference 11.5, *p* = .01 No significant between or within-group changes in MVPA from 6 to 12-months
Marcus (2021) [50] Marcus (2022) [49]	*N* = 199 Mean age: 43.8 ± 10.1 yrs Income: < $30,000/yr - 59% (*n* = 118)	Goal setting Self-monitoring Social support for PA Self-efficacy for PA	N/A	MVPA IG1 = enhanced intervention IG2 = original intervention (Marcus, 2013)	N/A	N/A	Significant increase in MVPA for IG1, unadj. median difference 28.0, *p* < .01, and IG2, unadj. median difference 16.0, *p* < .01, at 6-months No significant difference in MVPA change between IG1 and IG2 at 6-months No significant change in MVPA for IG1 or IG2 from 6 to 12-months (i.e., MVPA maintained) No significant difference in MVPA change between IG1 and IG2 from 6 to 12-months
Marshall (2013) [51]	*N* = 180 Mean age: 35.9 ± 8.7 yrs Income: < $18,000—61.0% (*n* = 110)	Self-monitoring Feedback Goal-setting Problem solving Social Support Self-efficacy Stress Reduction Familismo	N/A	MVPA IG1: Self-selected goals IG2: 10,000 steps/day IG3: 3,000 steps in 30 min./day	N/A	N/A	Significantly higher MVPA for IG3 vs. IG1 at 12-weeks, adj. median difference 7.0, *p* = .06 No other significant differences in MVPA at 12-weeks (*p*-value set at .1)
Salinas (2019) [52]	*N* = 620 Mean age: 40.4 ± 10.3 years Income: Not specified; labeled as low-income	N/A	N/A	MVPA IG vs CG (community health and safety)	N/A	N/A	No significant changes in MVPA for IG vs. CG at 16-weeks

*Note*. BCM = behavior change mechanism; PA = physical activity; MVPA = moderate-to-vigorous physical activity; IG = intervention group; CG = control group; adj. = adjusted; unadj. = unadjusted

Mean ages ranged from 28.4 [42] to 44.6 years old [43] and most studies included a wide age range for inclusion, 18–67 [52] and 18–65 years [40, 44, 46, 49, 51]. Only two studies limited the age range for inclusion, 18–40 [42] and 35–64 years [43], and one did not specify a range [41]. Sample sizes across the studies ranged from 35 [41] to 620 [52] with a total sample size of 2,303 women who self-identified as Hispanic/Latina. Approximately 78% (*n* = 1,801) of the total sample identified Mexico as their country of origin, 10.5% (*n* = 243) were from the Dominican Republic, Central and/or South America, 6.1% (*n* = 140) were from the United States/Puerto Rico, and 5.5% (*n* = 128) were from other/not specified. See Supplementary File 2 for a breakdown of Hispanic/Latina subgroups. Of note, the sample size information related to Hispanic/Latina subgroup data equaled 2,312 (100.4%) women rather than 2,303 due to inconsistencies in reporting *n*s that were greater than sample size *n*s [42, 49] and reporting subgroup percentages that totaled 103% rather than 100% [46].

### Physical activity measurements and outcomes

ActiGraph accelerometers were used to collect physical activity data in all studies [40–42, 44, 46, 49, 51, 52] except one, which used the Kenz Lifecorder Plus Accelerometer [43]. The physical activity outcomes included MVPA [40, 41, 44, 46, 49, 51, 52], moderate physical activity [43], moderate lifestyle, and moderate walking [42]. The cut-points used for accelerometry data processing included mostly Freedson 1998 [42, 44, 46, 49, 51, 52] and one study used Troiano 2008 [40]. Two studies did not explicitly specify the cut-points used [41, 43] but Koniak-Griffin et al. (2015) cited Freedson 1998 when describing accelerometer counts in the vertical plane. The accelerometer axial output was not specified in most of the included studies [40, 41, 44, 46, 49, 52] but two indicated the accelerometers were designed to measure in the vertical plane [42, 43] and one mentioned recording uniaxial accelerations [51]. Vector Magnitude was not specified in any of the studies.

### Data collection time points

Most studies conducted data collection immediately post-intervention [40, 41, 44, 46, 49, 51, 52] except two [42, 43], which delayed analysis to 6-months and again at 12-months for a 12-week intervention [42] and at 9-months for a 6-month intervention [43]. Four studies included an additional 6-month maintenance period post-intervention [44, 46, 49, 52] and all but one [52] reassessed MVPA changes at the end of the maintenance period. The three that evaluated MVPA changes at the end of the maintenance period did not conduct any further follow-up analysis to determine any sustained MVPA changes [45, 47, 50]. The only studies that included a follow-up analysis post-intervention were at 3-months [43], 4-months [41], and one at both 3- and 9-months [42] post-intervention.

### Health-related outcome variables

Two-thirds (*n* = 6) [40–42, 44, 46, 49] of the nine unique studies [43, 51, 52] explored changes in health-related outcomes in addition to MVPA. Body mass index, the most common, was included in four studies [40–43], followed by waist circumference [40, 42, 43], high-density lipoproteins, low-density lipoproteins, total cholesterol [41, 43, 49], depression [41, 42, 46], energy intake/diet [41–43] in three studies each, weight, fasting glucose [41, 43], and triglycerides [43, 49] in two studies each. Body fat percentage, waist-hip ratio, interleukin-6, interleukin-8, tumor necrosis factor alpha [42], blood pressure [43], hemoglobin A1C [49], and stress [46] were in one study each.

### Intervention characteristics

Previously published literature was used to obtain further details when complete intervention characteristics were lacking in the included studies [53–56].

Theoretical Framework.

Five interventions were developed using a combination of at least two different theoretical frameworks [43, 44, 46, 49, 51], three used one theory [40–42], and one did not specify if one was used [52]. The theoretical frameworks included the Transtheoretical Model, Social Cognitive Theory, Stage of Motivational Readiness for Physical Activity [44, 46, 49], Community Prevention Model, Community-based Participatory Conceptual Framework [43], the Behavioral and Socioecological Models for Latino Health Promotion and Communication-Persuasion Model [51], Ecological Framework [40], Self-Determination Theory [41], and Social Support Framework [42]. See Supplementary File 3 for intervention characteristics by study.

#### Cultural-relevancy

All interventions were culturally-tailored and/or designed to be relevant for Hispanic women through formative research using focus groups and/or feedback from Latina/Hispanic women [44, 46, 49, 56], stakeholders [41], and/or community advisory groups [52, 54]. One study indicated community-based participatory research was implemented to design the intervention but did not specify who participated [51]. A culturally-relevant intervention that was developed by the National Heart, Lung, and Blood Institute was implemented in one study [43].

#### Intervention language

Four interventions were provided in Spanish [43, 44, 46, 51], two in English and Spanish [42, 49], and three did not specify the language of the intervention [40, 41, 52]. One study began with Spanish-only in year one and in year two provided the intervention in both Spanish and English [49]; therefore, we included this study findings in the Spanish and English category. Of the three studies that did not specify the intervention language, two of the interventions included bilingual promotoras [40, 41]. None of the interventions were provided only in English. See Supplementary File 4 for significant improvements in MVPA based on intervention language.

#### Intervention duration

Intervention duration ranged from 8-weeks [41] to 24-months [40], and four studies included an additional maintenance period of 6-months after the intervention [44, 46, 49, 52]. The mean duration of the interventions was 28.44 ± 29.25 weeks and 40.0 ± 30.25 weeks with the intervention maintenance periods included. When only including interventions with significant improvements in MVPA at any timepoint, mean intervention duration was 18.33 ± 8.52 weeks and 31.33 ± 22.69 weeks with the maintenance period included. See Supplementary File 5 for significant improvements in MVPA by intervention duration.

#### Intervention format

The majority of interventions (88.9%) used a combination of delivery formats (i.e., in-person, internet, telephone, text, email, print, DVD) [40–44, 46, 49, 51] and ranged from a single format intervention (i.e., in-person) [52] to four [43, 46, 49] different formats. All studies had some type of in-person session with 66.7% of those demonstrating significant improvements in MVPA [41, 42, 44, 46, 49, 51]; however, only 50% [41, 42, 51] of the six interventions [40, 43, 52] that were primarily delivered in-person significantly improved MVPA. Two interventions were primarily mail-based print [44, 49] and one was internet-based (website) [46] with all three demonstrating significant improvements in MVPA and including one in-person session at the start of the intervention. Promotora-led interventions only significantly increased MVPA 50% of the time [41, 42, 51] (*n* = 3 of 6) [40, 43, 52]. See Supplementary File 6 includes a table for significant MVPA improvements by intervention format. This table includes the number of interventions that had a primary delivery route via in-person, mail-based print, or internet-based. For the format category of in-person any amount, in-person any individual, and in-person any group, these apply to interventions that include any amount of an intervention component delivered in that format. Interventions were included in the print, telephone, email, or text format categories if the intervention delivered a component in this format regardless of the amount.

### Moderate-to-vigorous physical activity

#### Preliminary findings

One study included preliminary analysis at 12-months for a 24-month intervention [39] and the intervention significantly improved MVPA at 12-months; however, the 12-month findings were not included in the overall narrative synthesis of findings because the primary intervention was still ongoing.

#### Shorter-term findings

Five [42, 44, 46, 49, 51] of nine studies [40, 41, 43, 52] (55.6%) demonstrated significant improvements in MVPA at the end of the primary intervention, including for both IG groups [49], one IG compared to another IG [51], and compared to the CG [42, 44, 46]. Two of these studies did not assess MVPA changes until 3-months after the end of the intervention [42, 43].

#### Longer-term findings

All five studies with follow-up analyses demonstrated significant improvements in MVPA for the IG [41] and IG compared to CG [42, 45, 47]. The fifth study did not demonstrate significant changes in MVPA from 6- to 12-months for both IG1 and IG2 [50]; however, this indicated that the increased MVPA at 6-months was sustained at 12-months. Three of these studies assessed MVPA change at the end of a 6-month maintenance period that followed a 6-month intervention [45, 47, 50] and two assessed MVPA changes at 4-months [41] and 9-months [42] post-intervention. One study provided conflicting *p*-values, *p* < 0.02 in the text and *p* = 0.291 in a table; however, it was included as significant findings because the authors discussed significant improvements in MVPA in the body of the manuscript [41]. Multiple attempts were made to contact the author without a response.

## Socioecological levels targeted

The majority of studies (66.7%) targeted three socioecological levels with a range of three [43, 44, 46, 49, 51, 52] to five [40] (*M* = 3.44 ± 0.73). For interventions that targeted the neighborhood/environmental level, MVPA significantly improved in 83.3% of studies [41, 42, 44, 46, 49] and 66.7% of studies that targeted the intrapersonal and interpersonal levels demonstrated significant improvements [41, 42, 44, 46, 49, 51]. Only 50% of interventions that targeted the organizational level had significant improvements in MVPA [41, 42, 51]. See Table 2 for significant MVPA improvements by socioecological level.

**Table 2:** Socioecological Level Targeted by Study

Author (Year)	Intrapersonal	Interpersonal	Organizational	Neighborhood/ Environment	Policy	Total Levels	ST Findings	LT Findings
Arredondo (2022) [40]	1	1	1	1	1	5	-	N/A
Cherrington (2015) [41]	1	1	1	1	0	4	-	+
Keller (2014) [42]	1	1	1	1	0	4	+	+
Koniak-Griffin (2015) [43]	1	1	1	0	0	3	-	N/A
Marcus (2013) [44]	1	1	0	1	0	3	+	N/A
Marcus (2016) [45]	1	1	0	1	0	3	+	+
Marcus (2022) [49]	1	1	0	1	0	3	+	+ *
Marshall (2013) [51]	1	1	1	0	0	3	+	N/A
Salinas (2019) [52]	1	1	1	0	0	3	-	N/A
Significant *n* / Total *n*	6/9	6/9	3/6	5/6	0/1			

*Note.* 1 = Socioecological level targeted; 0 = socioecological level not targeted;— = no significant increase in moderate-to-vigorous physical activity; +  = significant increase in moderate-to-vigorous physical activity; N/A = not applicable; * = findings not significant but demonstrated significant increase in moderate-to-vigorous physical activity was maintained from 6- to 12-months

### Behavior change mechanisms

Eight unique studies (88.9%) specified which BCM were targeted by the intervention [40–44, 46, 49, 51] for a total of 23 different BCM, and only one study did not specify BCM [52]. Four studies (44.4%) measured and tested for changes in BCM [40, 42, 44, 46] which included a total of 14 unique BCM. Only two studies (22.2%) conducted mediation analyses to determine which BCM mediated MVPA improvements [44, 46]. See Table 3 for BCM by study and Table 4 for findings by BCM.

**Table 3:** Behavior Change Mechanisms by Study

Author Year	BCM Targeted	BCM Change	Mediation Analysis	Increased MVPA
Arredondo [40] 2022	Behavioral strategies for PA	+	N/A	-
Cherrington 2015 [41]	Autonomy	N/A	N/A	+
	Perceived competence	N/A	N/A	+
	Relatedness to others	N/A	N/A	+
Keller 2014 [42]	Social support for exercise	+ /*	N/A	+
	General social support overall	-	N/A	+
	Emotional/informational	**	N/A	+
	Tangible	**	N/A	+
	Affectionate	-	N/A	+
	Positive social interactions	-	N/A	+
Koniak-Griffin 2015 [43]	Goal-setting Self-monitoring	N/A N/A	N/A N/A	- -
Marcus 2013 [44]	Self-efficacy for PA	+	N/A	+
	Cognitive processes	+	N/A	+
Marquez 2016 [45]	Behavioral processes	+	N/A	+
	Social support for exercise:			
	***Family participation***	** + **	± ***	** + **
	Family rewards & punishments	+	-	+
	Friend participation	+	-	+
Marcus 2016 [46]	***Self-efficacy for PA***	** + **	** + **	** + **
	Cognitive processes	+	-	+
Hartmann 2017 [47]	Behavioral processes	+	-	+
	PA enjoyment	-	-	+
Larsen 2021 [48]	Social support for exercise:			
	Family participation	-	N/A	+
	Family rewards & punishments	-	N/A	+
	Friend participation	-	N/A	+
Marcus 2021, 2022 [49, 50]	Goal-setting	N/A	N/A	+
	Self-monitoring	N/A	N/A	+
	Social support for PA	N/A	N/A	+
	Self-efficacy	N/A	N/A	+
Marshall 2013 [51]	Self-monitoring	N/A	N/A	+
	Feedback	N/A	N/A	+
	Goal-setting	N/A	N/A	+
	Problem solving Social support	N/A N/A	N/A N/A	+ +
	Self-efficacy	N/A	N/A	+
	Stress reduction	N/A	N/A	+
	Familismo	N/A	N/A	+
Salinas 2019 [52]	Not specified	N/A	N/A	-

***Note.*** BCM = behavior change mechanism; +  = statistically significant; N/A = not applicable;—= not statistically significant; * = significant increase for intervention group at 6-months and significant decrease for intervention vs. control from 6- to 12-months; ** = significantly higher for control vs. intervention; *** = statistically significant at 6-months, not statistically significant at 12-months

**Table 4:** Significant Findings by Behavior Change Mechanism

Behavior Change Mechanism	*n* Studies BCM Included	*n* Studies BCM ∆ Tested	*n* Studies BCM ∆ Significant	*n* Studies Mediation Analysis	*n* Studies Mediation Significant	*n* Studies MVPA ↑ Significant
Self-efficacy for PA/self-efficacy	4	2	2	1	1	4
Cognitive processes	2	2	2	1	0	2
Behavioral processes	2	2	2	1	0	2
PA enjoyment	1	1	0	1	0	1
SSE family participation	2	2	1	1	1	2
SSE family rewards & punishments	2	2	1	1	0	2
SSE friend participation	2	2	1	1	0	2
SSE/Social support for PA	3	1	1*	N/A	N/A	3
General social support overall	1	1	0	N/A	N/A	1
Emotional/informational	1	1	1**	N/A	N/A	1
Tangible	1	1	1**	N/A	N/A	1
Affectionate	1	1	0	N/A	N/A	1
Positive social interactions	1	1	0	N/A	N/A	1
Behavioral strategies for PA	1	1	1	N/A	N/A	0
Goal-setting	3	0	N/A	N/A	N/A	2
Self-monitoring	3	0	N/A	N/A	N/A	2
Autonomy	1	0	N/A	N/A	N/A	1
Perceived confidence	1	0	N/A	N/A	N/A	1
Feedback	1	0	N/A	N/A	N/A	1
Problem solving	1	0	N/A	N/A	N/A	1
Stress reduction	1	0	N/A	N/A	N/A	1
Relatedness to others	1	0	N/A	N/A	N/A	1
Familismo	1	0	N/A	N/A	N/A	1

***Note***. BCM = behavior change mechanism; ∆ = change; MVPA = moderate-to-vigorous physical activity; PA = physical activity; SSE = social support for exercise; * = significant for intervention group at 6-months and significant decrease for intervention group vs. control group at 12-months; ** = significantly higher for control group vs. intervention group; N/A = not applicable

#### Behavior change mechanisms and socioecological levels

Self-efficacy [48] at the intrapersonal level and family participation [45] at the interpersonal level were the only BCM that mediated improvements in MVPA at 6-months. Only one study tested for mediation again at 12-months which found family participation no longer mediated improvements of MVPA [45]. Both studies that included a mediation analysis implemented a 6-month intervention with a 6-month maintenance period. Cognitive processes, behavioral processes, physical activity enjoyment [48], family rewards and punishment, and friend participation [45] did not mediate MVPA improvements in the one mediation analysis conducted for each concept.

### Behavior change techniques

Table 5 describes the BCT implemented at each socioecological level for interventions that mediated improvements in MVPA and/or demonstrated significant improvements in MVPA. All interventions included a variety of BCT that targeted BCM at the intrapersonal and interpersonal levels described above [40–44, 46, 49, 51, 52]. Only self-efficacy [46] and family participation [45] were found to mediate MVPA; therefore, the BCT implemented for self-efficacy at the intrapersonal level and family participation at the interpersonal level in those interventions are detailed in Table 4. Six interventions targeted the organizational level, which included group physical activity sessions [40, 42, 43, 52] and/or individual/group discussions [41–43, 51, 52] at community-based organizations. Only three (50%) of those demonstrated significant improvements in MVPA [41, 42, 51]; therefore, the BCT at the organizational level were not included in Table 5. Of the BCT at this level that demonstrated significant findings, only one provided group physical activity sessions [42] and the other two provided individual and/or group discussions [41, 51].

**Table 5:** Effective Behavior Change Techniques by Socioecological Model Level

Concept	Behavior Change Mechanism/ Intervention Target	Behavior Change Technique
***Intrapersonal***		
Self-efficacy for exercise	Self-efficacy	• Completed small attainable goals, provided feedback towards goals, implemented problem solving around barriers [48] • Provided one health coaching session with support using motivational interviewing to increase confidence [48] • Provided tailored reports based on self-efficacy [48]
***Interpersonal***		
Social support for exercise	Family participation	• Addressed barriers including childcare and partner support [44] • Information on social support including to identify individuals who support versus sabotage physical activity goals, elicit and enlist help from diverse sources, benefits of exercise partners [45]
***Neighborhood/Environment***		
Opportunity	Physical activity access	• Identified physical activity-related community resources and activities for families [45] • Provided online maps with 1–3 mile walking routes in neighborhoods [46] • Provided individual report mapping physical activity locations (walking routes, gyms, parks) near participants’ homes [49] • Maps with walking safe routes, historic facts and local lore of interest in neighborhoods [42]
Neighborhood safety	Physical activity access	• Provided DVD with home exercises when perceived neighborhood safety as a barrier [41] • Addressed neighborhood safety concerns with alternatives to outdoor physical activity [48] • Provided physical activity tip sheets that addressed barriers to physical activity in Latinas (e.g., neighborhood safety) [49]
Environmental barriers	Physical activity access	• Problem solved inclement weather [44] • Provided individually tailored reports addressing different levels of environmental factors affecting physical activity [49]

Five of the six (83.3%) interventions that included BCT targeting the neighborhood/environmental level demonstrated significant improvements in MVPA [41, 42, 44, 46, 49]. These BCT addressed access to physical activity (i.e., opportunity) and solutions to overcome physical activity barriers (i.e., safety, environmental) by providing online or printed walking routes/maps [42, 46, 49], community/neighborhood physical activity resources [44], alternatives to outdoor physical activity [41, 46, 49], and methods to overcome neighborhood safety issues [41, 42, 46, 49]. Only one intervention addressed the policy level but did not significantly increase MVPA, which included a BCT where promotoras advocated for changes to barriers in the neighborhood environment. Details regarding the implementation of any changes were not provided [40].

### Proposed conceptual model

**Fig. 2:**
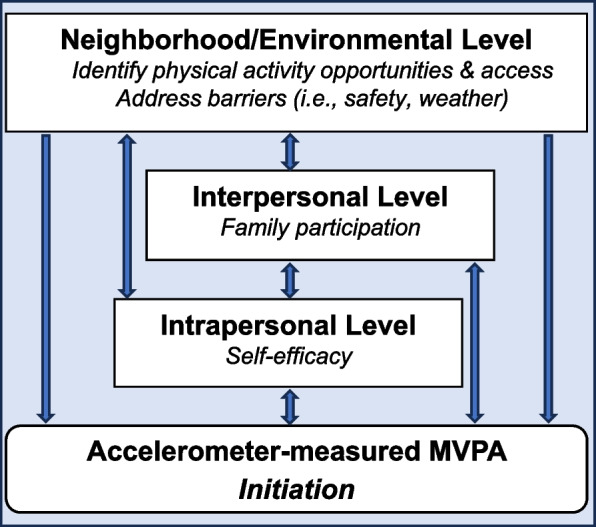
Proposed conceptual model of a multilevel physical activity intervention for Hispanic women. *Note*. MVPA = moderate-to-vigorous physical activity

A conceptual model of a multilevel intervention to initiate participation in accelerometer-measured MVPA for Hispanic women was developed based on the narrative synthesis findings (Fig. 2). The model proposes that interventions targeting self-efficacy at the *intra*personal level, family participation at the *inter*personal level, and physical activity opportunities and access at the neighborhood/environmental level, including methods to overcome barriers (e.g., safety, weather), may promote successful initiation of accelerometer-measured MVPA among Hispanic women. The conceptual model specifies MVPA initiation because all interventions that significantly increased MVPA were evaluated immediately at the conclusion of the intervention [44, 46, 49, 51] or intervention maintenance period [45, 47, 50] except two [41, 42]. The longest post-intervention evaluation was at 4-months [41] and 9-months [42] after the end of the intervention.

### Risk of bias and grading of recommendations assessment, development, and evaluation

The majority of studies were at low risk of bias [40, 42–44, 46, 49], one had some concerns [51, 52], and one had moderate [41] risk of bias. See Supplementary File 7 for risk of bias ratings by domain for each study.

Each GRADE domain was rated as follows: 1) methodological limitations of the studies – no concerns; 2) indirectness – serious concerns; 3) imprecision – serious concerns, borderline; 4) inconsistency – no concerns; and 5) publication bias – no concerns, borderline. See Supplementary File 8 for a narrative description of each domain judgement. Based on GRADE, there was moderate to low quality evidence that physical activity interventions likely resulted in an increase in MVPA among Hispanic women [36, 38].

## Discussion

This comprehensive systematic review was conducted to assess interventions to improve accelerometer-measured MVPA among samples of Hispanic women and determine the socioecological levels, BCM, and BCT to increase accelerometer-measured MVPA. A narrative synthesis was conducted and a conceptual model was developed and proposed that details a multilevel intervention for Hispanic women to initiate accelerometer-measured MVPA participation. A total of 14 studies were identified that met inclusion and exclusion criteria, nine of which were unique interventions and five studies provided additional findings.

### Accelerometer-measured moderate and moderate-to-vigorous physical activity

Overall, the majority (66.7%) of intervention studies reviewed were successful at improving accelerometer-measured moderate and/or MVPA among Hispanic women, either within the intervention groups or for the intervention compared to control or comparison groups. Of the six unique studies that compared an intervention with a control group [40, 42–44, 46, 52], 50% demonstrated significantly higher accelerometer-measured moderate and/or MVPA [42, 44, 46]. The one study without a control or comparator group demonstrated significant MVPA improvements [41]. Two MVPA interventions [49] were compared in one study and both interventions significantly increased accelerometer-measured MVPA; however, no differences were found between the two interventions. The one study that compared three intervention arms based on the step goal implemented demonstrated significantly higher accelerometer-measured MVPA for one of the interventions arms compared to the other two [51].

The overall findings that a majority of interventions increased accelerometer-measured MVPA among the studies included in this review align with previous reviews conducted that evaluated physical activity interventions among ethnically and racially diverse women [16], adults [15], and children [18]. However, the findings are contrary to the one review of physical activity interventions implemented among primarily Hispanic women [11]. The inconsistency between the findings of this review and the review that included primarily Hispanic women may be due to a few reasons. The intervention studies included in the previous review included mostly subjective physical activity measures potentially limiting validity and/or reliability of findings, focused on changing behavioral variables primarily at the intrapersonal level, and/or most interventions were designed without any theoretical guidance [11].

It is important to note that the improvements in MVPA demonstrated in this review were mostly measured in a shorter-term period, at the conclusion of the interventions and/or maintenance periods (i.e., MVPA initiation), consistent with previous reviews synthesizing the physical activity literature [11, 14, 16, 17, 21, 22]. Only one study reassessed accelerometer-measured MVPA at a longer-term interval which was at nine-months post-intervention [42]. It is an imperative that future investigations testing the efficacy of physical activity interventions among Hispanic women include longer-term assessments of outcomes to determine the sustainability of accelerometer-measured MVPA improvements (i.e., MVPA maintenance).

### Socioecological levels

Interventions that targeted the intrapersonal [48], interpersonal [45], and neighborhood levels [41, 42, 44, 46, 49] demonstrated efficacy at increasing accelerometer-measured MVPA among Hispanic women. This review did not provide evidence to support targeting the organizational (*n* = 3 of 6 interventions) or policy (*n* = 0 of 1 intervention) levels to increase MVPA among Hispanic women; however, evidence targeting the policy level was scant.

### Behavior change mechanisms

Few studies included in this review tested for changes in BCM [40, 42, 44, 46] and even fewer included mediation analyses to determine which BCM were responsible for increases in accelerometer-measured MVPA [45, 48] consistent with previous reviews of physical activity interventions [14]. Only self-efficacy and family participation were found to mediate accelerometer-measured MVPA; however, these effects were found immediately post-intervention and before the end of the 6-month maintenance period for both interventions [45, 48]. Self-efficacy was not re-analyzed to determine if mediation was sustained following the maintenance period in one study [48] and family participation no longer mediated accelerometer-measured MVPA at the conclusion of the 6-month maintenance period in the other [45]. These findings suggest that self-efficacy and family participation may be responsible for the initiation of Hispanic women’s MVPA, conflicting with previous research with inconclusive findings related to self-efficacy and social support as BCM [14]. Potential explanations for the contradictory findings may be due to inadequate measures of self-efficacy and social support, incorrect BCT used to target the BCM, inadequate power to detect changes, and/or use of mostly subjective measures of physical activity outcomes [14].

It is important to highlight the evidence available to determine the BCM responsible for accelerometer-measured MVPA initiation among Hispanic women was extremely limited [45, 48] and nonexistent related to maintenance of accelerometer-measured MVPA. Future research should be powered to test physical activity interventions in the shorter- and longer-term to confirm and/or determine additional BCM responsible for both accelerometer-measured MVPA initiation and maintenance among Hispanic women. Otherwise, it will continue to be impossible to determine how or why interventions do or do not work, which is necessary to streamline interventions to target only the necessary BCM [7, 13].

### Behavior change techniques

The BCT implemented to increase self-efficacy at the intrapersonal level included goal-setting with feedback, problem solving, coaching to increase confidence, and tailored reports based on self-efficacy level [48]. The BCT implemented to increase family participation at the interpersonal level addressed barriers to physical activity (e.g., childcare and partner support), encouraged individuals to identify a person supportive of physical activity, and provided information about the benefits of exercise partners [45]. The BCT at the neighborhood/environmental level that increased MVPA identified specific opportunities for physical activity in neighborhoods surrounding participants homes, including when neighborhood safety and environmental barriers (e.g., weather) existed. The one study out of four that included group physical activity sessions at community organizations and increased MVPA also identified opportunities at the neighborhood level for physical activity [42], suggesting that providing resources that identify physical activity opportunities may be more beneficial at improving MVPA compared to hosting in-person group physical activity sessions.

Based on the findings of this systematic review, a proposed conceptual model of a multilevel intervention to initiate participation in accelerometer-measured MVPA for Hispanic women was presented in Fig. 2. This conceptual model builds upon previous reviews [11, 14–18, 21–23, 28] by providing intervention targets and BCT at the intrapersonal, interpersonal, and neighborhood/environmental level that have been found to improve Hispanic women’s accelerometer-measured MVPA. Future MVPA interventions should be designed guided by this conceptual model and tested to confirm and/or extend the findings based on this review.

### Intervention characteristics

MVPA interventions should be provided in both English and Spanish and allow participants to choose their preferred language. The mean intervention duration among interventions successful at increasing accelerometer-measured MVPA was 18 weeks and 31 weeks when intervention maintenance periods were included. These are both longer than the median length of 3-months identified in a previous review of interventions implemented among African American and Hispanic women [16], but provide a starting point to inform intervention duration specific to Hispanic women considering previous recommendations have been inconclusive [11]. The three interventions lasting 6-months with a 6-month maintenance period [44, 46, 49], two 12-week interventions [42, 51] and one 8-week intervention [41] all significantly increased MVPA. Interventions shorter than 6-months may be beneficial but the sample sizes were fairly small [41, 42] and most studies only determined MVPA changes immediately after the intervention and/or maintenance periods ended [44, 46, 49, 51]. These factors limit the ability to draw strong conclusions related to sustainability of effects, warranting further research with longer term follow-up.

Mail-based print or internet-based interventions with limited in-person individual sessions that focused on goal-setting increased accelerometer-measured MVPA more often compared to primarily in-person format with group sessions. Telephone calls and/or text messages were also beneficial in augmenting interventions. Across the study samples with income data, more than 50% of each had an income of less than $30,000 [40, 42–44, 49, 51], and approximately 69% of the experimental group testing the internet-based intervention also had an annual household income < $30,000 [46]. These findings indicate that internet-based interventions may be feasible in lower income Hispanic women and also extend previous research demonstrating that electronic and mobile health interventions had efficacy increasing African American and Hispanic women’s subjectively-measured physical activity [16]. Interestingly, promotoras did not appear to influence MVPA improvements. Future investigations should focus on formative research to corroborate these findings with samples of Hispanic women and determine the contextual-relevancy and utility of mail- and/or internet-based MVPA interventions with limited in-person contact. Additional randomized trials should be conducted to compare intervention effects between formats to determine the best intervention delivery method specific to Hispanic women.

Physical activity interventions have not been designed to be contextually-appropriate to any specific age group of Hispanic women. Most studies in this review included a wide range of ages for inclusion [40, 41, 44, 46, 49, 51, 52]. The two studies that did restrict the inclusion age included ages of women across life stages (e.g., young adult, adult, and middle aged) [42, 43]. The uptake and sustainability of behavioral interventions are context dependent [7]; therefore, they should consider the needs of Hispanic women specific to their age to maximize relevancy. For example, the needs of early adult/adult aged women caring for young children are different than those of middle aged who may have independent or grown children but care for aging parents or grandchildren [57]. MVPA interventions should be designed and tested to determine if the identified BCM and BCT at the specific socioecological levels increase accelerometer-measured MVPA among specific age groups of Hispanic women.

Cardiovascular disease is the second leading cause of death in Hispanic women [5], yet investigation into the impact of MVPA interventions on secondary cardiovascular outcomes was limited. Research demonstrates that meeting the national MVPA guidelines of 150 min per week improves cardiovascular risk factors (e.g., blood pressure, lipids, fasting glucose, depression) and there is a dose response between MVPA and cardiovascular risk factors [1]. However, there is insufficient evidence for MVPA recommendations to be specific to any sex, race, ethnicity, or age group [1]. This demonstrates the need to explore the relationship between MVPA and cardiovascular risk factors among Hispanic women.

### Strengths/limitations

This is the first review conducted investigating intervention efficacy on accelerometer-measured MVPA, or the socioecological levels, BCM, and BCT to increase accelerometer-measured MVPA in a large sample of Hispanic women. The majority of the women originated from Mexico allowing for some generalizability of findings to Mexican and Mexican–American women living in the United States but not necessarily to women from other Spanish-speaking countries. The inclusion criteria across the studies in this review were wide demonstrating that the interventions were not designed to be contextually-relevant to any specific age group or life stage; however, the mean ages across studies did fall into the adult age group (i.e., 25 to 44 years) which allows for some generalizability of findings to this group of Hispanic women. All intervention studies included in this review included accelerometer-measured MVPA as the outcome of study increasing the validity of the review findings.

There are limitations of this review that also need to be noted. A meta-analysis was not conducted due to the high heterogeneity across studies (i.e., study designs, intervention dose, length and delivery format, outcome measure assessment schedules, and statistical analysis plans) which limit the interpretation of meta-analytic findings [58, 59]. Using GRADE, the quality of evidence for interventions to increase MVPA was moderate to low due to serious concerns for indirectness, borderline serious concerns for imprecision, and borderline concerns for publication bias. However, both significant and not significant studies were identified and included, reducing the likelihood that studies without significant findings were not published. The limited number of studies that conducted formal mediation analyses prevents strong conclusions from being drawn. However, this review does provide initial evidence related to the BCM responsible for MVPA among Hispanic women advancing behavior change science. None of the included studies conducted multilevel modeling to determine intervention effects at higher levels of the socioecological model (i.e., organizational, neighborhood/environmental, policy), preventing causal relationships from being determined. The studies included in this review did not assess longer term changes in MVPA, limiting the ability to draw conclusions related to the sustainability of MVPA (i.e., maintenance) from the interventions. Finally, some domains of MVPA may not be accurately captured using accelerometers (e.g., housework, gardening).

### Implications

Future MVPA interventions should be developed to target self-efficacy and family participation and identify opportunities for physical activity near their homes including methods to overcome neighborhood safety and environmental-related barriers for Hispanic women in specific age groups. The identification of BCM responsible for Hispanic women’s MVPA maintenance was not possible because of the limited research available, necessitating additional research. Interventions should be designed and powered to conduct mediation analyses and/or multilevel modeling to determine the specific BCM and socioecological level targets responsible for accelerometer-measured MVPA improvements in both the shorter- and longer-term. Research investigating intervention characteristics should be conducted to determine if interventions lasting 18 to 31 weeks (with maintenance period) delivered via mail-based print and the internet increases MVPA. The exploration of changes in cardiovascular risk factors secondary to increased MVPA among Hispanic women is warranted.

Practitioners should assess and discuss MVPA at each visit with Hispanic women, including setting MVPA goals and progress towards goals, problem-solving barriers, encouraging family participation, and identifying neighborhood resources for physical activity. Policymakers should ensure comprehensive lists of physical activity resources and activities in neighborhoods/communities are developed and available to residents. Funders should increase the availability of additional financial resources for investigators to conduct longer-term follow-up evaluations of interventions to determine the sustainability of MVPA improvements.

## Conclusion

Self-efficacy at the intrapersonal level and family participation at the interpersonal level of the socioecological model were the only BCM found to mediate MVPA initiation among Hispanic women. Interventions that included BCT targeting confidence, goals, problem solving at the intrapersonal level, partner support and overcoming childcare barriers at the interpersonal level, and physical activity opportunities and methods to overcome barriers (e.g., safety, weather) at the neighborhood/environmental level significantly increased MVPA. Multilevel MVPA interventions should be offered in a choice of English or Spanish and designed according to the developed conceptual framework. In addition, interventions should last approximately 18-weeks with an additional 12-week maintenance period via mail- or internet-based format with limited in-person sessions. Interventions should be tested to determine change in MVPA among Hispanic women in specific age groups and explore effects on cardiovascular health. Future research should be conducted to determine additional BCM and BCT necessary to initiate and maintain Hispanic women’s accelerometer-measured MVPA participation.

## Supplementary Information

Supplementary Material 1.

Supplementary Material 2.

Supplementary Material 3.

Supplementary Material 4.

Supplementary Material 5.

Supplementary Material 6.

Supplementary Material 7.

Supplementary Material 8.

## Data Availability

All data generated or analyzed during this study are included in this published article and its supplementary information files.

## Abbreviations

MVPA: Moderate to vigorous physical activity
BCM: Behavior change mechanisms
BCT: Behavior change techniques
