# Supplementary material for: The socioecological model levels, behavior change mechanisms, and behavior change techniques to improve accelerometer-measured physical activity among Hispanic women: a systematic review
Source: Int J Behav Nutr Phys Act. 2025 Jun 19;22:80. doi: 10.1186/s12966-025-01783-y (PMC12180251; doi:10.1186/s12966-025-01783-y)
Supplement: Supplementary file 1 — Supplementary Material 1. [file 12966_2025_1783_MOESM1_ESM.docx]

| **Supplementary File 1.** Search Strategy | | |
| --- | --- | --- |
| Database | Search Strategy | Records |
| Scopus | (TITLE-ABS-KEY ((hispanic* OR "mexican american*" OR latin OR latinx OR latina)) AND TITLE-ABS-KEY ((exercis* OR "physical activit*")) AND TITLE-ABS-KEY ((women OR woman OR ladies OR female*)) AND TITLE-ABS-KEY (adult)) AND (LIMIT-TO (LANGUAGE, "english")) | 3637 |
| PubMed | ((((("hispanic or latino"[MeSH Terms] OR ("hispanic"[All Fields] AND "or"[All Fields] AND "latino"[All Fields]) OR "hispanic or latino"[All Fields] OR "hispanic"[All Fields] OR "hispanics"[All Fields]) AND ("womans"[All Fields] OR "women"[MeSH Terms] OR "women"[All Fields] OR "woman"[All Fields] OR "women s"[All Fields] OR "womens"[All Fields])) OR (("hispanic or latino"[MeSH Terms] OR ("hispanic"[All Fields] AND "or"[All Fields] AND "latino"[All Fields]) OR "hispanic or latino"[All Fields] OR "hispanic"[All Fields] OR "hispanics"[All Fields] OR ("hispanic or latino"[MeSH Terms] OR ("hispanic"[All Fields] AND "or"[All Fields] AND "latino"[All Fields]) OR "hispanic or latino"[All Fields] OR "latino"[All Fields] OR "latinos"[All Fields]) OR ("latinx"[All Fields] OR "latinxs"[All Fields]) OR ("hispanic or latino"[MeSH Terms] OR ("hispanic"[All Fields] AND "or"[All Fields] AND "latino"[All Fields]) OR "hispanic or latino"[All Fields] OR "latina"[All Fields] OR "latinas"[All Fields] OR "latina s"[All Fields]) OR ("mexican americans"[MeSH Terms] OR ("mexican"[All Fields] AND "americans"[All Fields]) OR "mexican americans"[All Fields] OR ("mexican"[All Fields] AND "american"[All Fields]) OR "mexican american"[All Fields])) AND ("femal"[All Fields] OR "female"[MeSH Terms] OR "female"[All Fields] OR "females"[All Fields] OR "female s"[All Fields] OR "femals"[All Fields]))) AND ("Exercise"[MeSH Terms] OR ("Exercise"[MeSH Terms] OR "Exercise"[All Fields] OR ("physical"[All Fields] AND "activity"[All Fields]) OR "physical activity"[All Fields])) AND "adult"[MeSH Terms]) NOT ("pregnan*"[All Fields] AND "adult"[MeSH Terms])) AND (alladult[Filter]) | 2469 |
| CINAHL | S1 (MH "Hispanic Americans+") OR "hispanic"  S2 "LATIN*"  S3 (MH "Mexican Americans") OR "MEXICAN AMERICAN*"  S4 S1 OR S2 OR S3  S5 (MH "Exercise+") OR "EXERCISE"  S6 (MH "Physical Activity") OR "PHYSICAL ACTIVITY"  S7 S5 OR S6  S8 S4 AND S7  S9 S4 AND S7  S10 S4 AND S7  S11 S4 AND S7  S12 S4 AND S7  S13 S4 AND S7 | 1282 |
| PsychINFO | 1. ((hispanic* or HISPANIC AMERICAN* or MEXICAN AMERICAN* or LATINO or LATINX) and (EXERCIS* or PHYSICAL ACTIVIT*)).mp. [mp=title, abstract, heading word, table of contents, key concepts, original title, tests & measures, mesh word] 2. limit 1 to adulthood <18+ years> 3. limit 2 to (english language and female) | 1086 |
| Cochrane Library | #1 hispanic* or HISPANIC AMERICAN* or MEXICAN AMERICAN* or LATINO or LATINX  #2 EXERCIS* or PHYSICAL ACTIVIT*  #3 #1 AND #2  #4 ADULT  #5 FEMALE  #6 #3 AND #4 AND #5  #7 pregnanc*  #8 #6 NOT #7 | 71 |
