# Supplementary material for: The socioecological model levels, behavior change mechanisms, and behavior change techniques to improve accelerometer-measured physical activity among Hispanic women: a systematic review
Source: Int J Behav Nutr Phys Act. 2025 Jun 19;22:80. doi: 10.1186/s12966-025-01783-y (PMC12180251; doi:10.1186/s12966-025-01783-y)
Supplement: Supplementary file 2 — Supplementary Material 2. [file 12966_2025_1783_MOESM2_ESM.docx]

| Supplementary File 2. Setting and Hispanic/Latina Subgroup by Study | | |
| --- | --- | --- |
| Author  (Year) | Setting | Hispanic/Latina  Subgroup |
| Arredondo  (2017) | San Diego County, CA USA | Mexico (*n*=394; 90.4%)  Not specified (*n*=42; 9.6%) |
| Cherrington  (2015) | Birmingham, AL  USA | Mexico (*n*=30, 86%)  El Salvador (*n*=3, 9%)  Costa Rica (*n*=2, 5%) |
| Keller  (2014)* | Southwest AZ  USA | Mexico (*n*=121, 85.8%)  United States (*n*=19, 13.5%)  Central America (*n*=1, 0.01%) |
| Koniak-  Griffin  (2015) | Los Angeles, CA  USA | Mexico (*n*=187, 83.9%)  Dominican, Central, South America  (*n*=32, 14.3%)  United States (*n*=4, 1.8%) |
| Marcus  (2013) | Providence, RI  USA | Dominican Republic (*n*=99, 37.3%)  Colombia (*n*=69, 26.0%)  Other (*n*=32, 12.1%)  Puerto Rico (*n*=28, 10.6%)  Guatemala (*n*=24, 9.1%)  Mexico (*n*=13, 4.9%) |
| Marcus  (2016)** | San Diego, CA  USA | Mexico (*n*=173; 84.4%)  Other (*n*=26; 12.7%)  Colombia (*n*=7; 3.4%)  Guatemala (*n*=2; 1.0%)  Puerto Rico (*n*=2; 1.0%)  Dominican Republic (*n*=1; 0.5%) |
| Marcus  (2022)* | San Diego, CA  USA | Mexico (*n*=177, 88.9%)  Other (*n*=19, 9.5%)  Colombia (*n*=2, 1%)  Cuba (*n*=1, 0.5%)  Puerto Rico (*n*=1, 0.5%)  Guatemala (*n*=1, 0.5%) |
| Marshall  (2013) | San Diego County, CA USA | Mexico (*n*=172, 95.6%)  United States (*n*=6, 3.1%)  Other (*n*=2, 1.3%) |
| Salinas  (2019) | Hidalgo County, TX USA | Mexico (*n*=534, 86.1%)  United States (*n*=80, 12.9%)  Other (*n*=6, 0.97%) |
| *Note*. * = total subgroup *n* > sample size *n*; ** = % per subgroup totaled 103%. | | |
