# Supplementary material for: The socioecological model levels, behavior change mechanisms, and behavior change techniques to improve accelerometer-measured physical activity among Hispanic women: a systematic review
Source: Int J Behav Nutr Phys Act. 2025 Jun 19;22:80. doi: 10.1186/s12966-025-01783-y (PMC12180251; doi:10.1186/s12966-025-01783-y)
Supplement: Supplementary file 3 — Supplementary Material 3. [file 12966_2025_1783_MOESM3_ESM.docx]

| **Supplementary File 3**. Intervention Characteristics | | | | | | |
| --- | --- | --- | --- | --- | --- | --- |
| Author  (year) | Theoretical  framework | Language | Delivery  format | Length | Time 1  MVPA  findings | Time 2  MVPA  findings |
| Arredondo  (2017)  Arredondo  (2022) | Ecological Framework for Health Behaviors | Not specified  (Bilingual Promotoras) | In-person  Group PA  (Church)  Telephone  Print  Promotoras | 24-months | +  12-months  (preliminary) | -  24-months |
| Cherrington  (2015) | Self-Determination Theory | Not specified  (Bilingual Promotoras) | In-person  Discussions  (Individual, Group)  PA DVD  Promotoras | 8-weeks | -  8-weeks | +  6-months |
| Keller  (2014) | Social Support Framework | Spanish &  English | In-person  Group PA  Discussions  (Individual, Spouse, Group)  Print  Promotoras | 12-weeks | +  6-months | +  12-months |
| Koniak-  Griffin  (2015) | Community Prevention Model  Community-Based Participatory Conceptual Framework | Spanish | In-person  Group PA  Discussions  (Individual, Group)  PA DVD  Telephone  Print  Promotoras | 6-months | -  9-months | N/A |
| Marcus  (2013)  Marquez  (2016) | Transtheoretical Model  Social Cognitive Theory  Stage of Motivational Readiness for Physical Activity | Spanish | Mail-based Print  Telephone  In-person  Discussions  (Individual) | 6-months  +  6-month maintenance | +  6-months | +  12-months |
| Marcus  (2016)  Hartman  (2017)  Larsen  (2021) | Transtheoretical Model  Social Cognitive Theory  Stage of Motivational Readiness for Physical Activity | Spanish | Internet-based (website, exercise videos)  Email  Telephone  In-person  Discussions  (Individual) | 6-months  +  6-month maintenance | +  6-months | +  12-months |
| Marcus  (2021)  Marcus  (2022) | Transtheoretical Model  Social Cognitive Theory  Stage of Motivational Readiness for Physical Activity | Spanish  (year 1)  Spanish or  English  (year 2) | Mail-based Print  Telephone  Text  In-person  Discussions  (Individual) | 6-months  +  6-month  maintenance | +  6-month | +  (MVPA maintained from 6 to 12-months) |
| Marshall  (2013) | Behavioral and Socioecological Models for Latino Health Promotion  Communication-Persuasion Model | Spanish | In-person  Discussions  (Group)  Print  Promotoras | 12-weeks | +  12-weeks | N/A |
| Salinas  (2019) | Not specified | Not specified | In-person  Group PA  Discussion/  education  (Individual, Group)  Promotoras | 16-weeks  +  6-month  maintenance | -  16-weeks | N/A |
| ***Note*.** (+) = significant improvement; (-) = not significant; N/A = not applicable | | | | | | |
