# Supplementary material for: The socioecological model levels, behavior change mechanisms, and behavior change techniques to improve accelerometer-measured physical activity among Hispanic women: a systematic review
Source: Int J Behav Nutr Phys Act. 2025 Jun 19;22:80. doi: 10.1186/s12966-025-01783-y (PMC12180251; doi:10.1186/s12966-025-01783-y)
Supplement: Supplementary file 4 — Supplementary Material 4. [file 12966_2025_1783_MOESM4_ESM.docx]

| **Supplementary File 4**. Significant Findings by Intervention Language | | |
| --- | --- | --- |
| Intervention Language | *n*  Studies | *n*  Significant  MVPA ↑ |
| Spanish | 4 | 3 |
| Spanish and English | 2 | 2 |
| Not specified | 3 | 1 |
| ***Note****.* MVPA = Moderate-to-vigorous physical activity. | | |
