# Supplementary material for: The socioecological model levels, behavior change mechanisms, and behavior change techniques to improve accelerometer-measured physical activity among Hispanic women: a systematic review
Source: Int J Behav Nutr Phys Act. 2025 Jun 19;22:80. doi: 10.1186/s12966-025-01783-y (PMC12180251; doi:10.1186/s12966-025-01783-y)
Supplement: Supplementary file 5 — Supplementary Material 5. [file 12966_2025_1783_MOESM5_ESM.docx]

| **Supplementary File 5**. Significant Findings by Intervention Duration | | |
| --- | --- | --- |
| Intervention Length | *n*  Studies | *n*  Significant MVPA ↑ |
| 24-months | 1 | 0 |
| 6-months | 1 | 0 |
| 6-months + 6-month maintenance | 3 | 3 |
| 16-weeks + 6-months maintenance | 1 | 0* |
| 12-weeks | 2 | 2 |
| 8-weeks | 1 | 1 |
| ***Note*.** MVPA = moderate-to-vigorous physical activity. * = this study did not reassess outcomes at the end of the maintenance period. | | |
