# Supplementary material for: The socioecological model levels, behavior change mechanisms, and behavior change techniques to improve accelerometer-measured physical activity among Hispanic women: a systematic review
Source: Int J Behav Nutr Phys Act. 2025 Jun 19;22:80. doi: 10.1186/s12966-025-01783-y (PMC12180251; doi:10.1186/s12966-025-01783-y)
Supplement: Supplementary file 6 — Supplementary Material 6. [file 12966_2025_1783_MOESM6_ESM.docx]

| **Supplementary File 6**. Significant Findings by Intervention Delivery Format | | |
| --- | --- | --- |
| Intervention Delivery Format | *n*  Studies | *n*  Significant  MVPA ↑ |
| In-person any amount | 9 | 6 |
| In-person primary delivery | 6 | 3 |
| In-person any individual | 6 | 5 |
| In-person any group | 6 | 3 |
| Print | 6 | 4 |
| Promotoras | 6 | 3 |
| Telephone | 5 | 3 |
| In-person group PA | 4 | 1 |
| DVD/internet-based PA | 3 | 2 |
| Mail-based print primary delivery | 2 | 2 |
| Internet-based primary delivery | 1 | 1 |
| Email | 1 | 1 |
| Text | 1 | 1 |
| ***Note.*** MVPA = moderate-to-vigorous physical activity; individual = discussions/motivational interviewing; any group = discussions and/or exercise; PA = physical activity. | | |
