# Supplementary material for: The socioecological model levels, behavior change mechanisms, and behavior change techniques to improve accelerometer-measured physical activity among Hispanic women: a systematic review
Source: Int J Behav Nutr Phys Act. 2025 Jun 19;22:80. doi: 10.1186/s12966-025-01783-y (PMC12180251; doi:10.1186/s12966-025-01783-y)
Supplement: Supplementary file 7 — Supplementary Material 7. [file 12966_2025_1783_MOESM7_ESM.docx]

| **Supplementary File 7.** Risk of Bias Assessment | | | | | | | | | |
| --- | --- | --- | --- | --- | --- | --- | --- | --- | --- |
| Authors  Year | Domain  1 | Domain  1b | Domain  2 | Domain  3 | Domain  4 | Domain  5 | Domain  6 | Domain 7 | Overall |
| ***RoB-2*** | | | | | | | | | |
| Keller  (2014) | Low | N/A | Low | Low | Low | Low | N/A | N/A | Low |
| Koniak-Griffin (2015) | Low | N/A | Low | Low | Low | Low | N/A | N/A | Low |
| Marcus  (2013) | Low | N/A | Low | Low | Low | Low | N/A | N/A | Low |
| Marcus  (2016) | Low | N/A | Low | Low | Low | Low | N/A | N/A | Low |
| Marcus  (2022) | Low | N/A | Low | Low | Low | Low | N/A | N/A | Low |
| ***RoB-2 CRT*** | | | | | | | | | |
| Arrendondo (2022) | Low | Low | Low | Low | Low | Low | N/A | N/A | Low |
| Marshall  (2013) | Low | Low | Low | Low | Low | Low | N/A | N/A | Low |
| Salinas  (2019) | Some  concerns | Low | Low | Low | Low | Low | N/A | N/A | Some concerns |
| ***ROBINS-I*** | | | | | | | | | |
| Cherrington (2015) | Low | N/A | Low | Low | Low | Moderate | Low | Low | Moderate |
| ***Notes.*** RoB-2 = Version 2 of the Cochrane Risk-of-Bias tool for randomized trials; ROB-2 CRT = the Revised Cochrane Risk-of-Bias tool for cluster-randomized trials; ROBINS-I = the Risk-of-Bias in Non-Randomized Studies of Interventions. N/A = not applicable. | | | | | | | | | |
