# Supplementary material for: The socioecological model levels, behavior change mechanisms, and behavior change techniques to improve accelerometer-measured physical activity among Hispanic women: a systematic review
Source: Int J Behav Nutr Phys Act. 2025 Jun 19;22:80. doi: 10.1186/s12966-025-01783-y (PMC12180251; doi:10.1186/s12966-025-01783-y)
Supplement: Supplementary file 8 — Supplementary Material 8. [file 12966_2025_1783_MOESM8_ESM.docx]

| **Supplementary File 8.** The Grading of Recommendations Assessment, Development, and Evaluation (GRADE) Domain Judgements | | |
| --- | --- | --- |
| **GRADE Domain** | **Judgement** | **Concerns about Certainty Domains** |
| Methodological limitations of the studies | Five of the intervention studies were RCTs, three Cluster RCTs, and one non-randomized trial. All were found to have a low risk of bias except two. One RCT had moderate RoB due to missing data but this was the smallest sample with only 35 participants (41). One of the cluster RCTs had some concerns arising from the randomization process in that participants group allocation was not concealed. However, participants did not know the status of other community centers (52). This domain was judged as no concerns. | No concerns |
| Indirectness | All studies included only Hispanic women, outcome measures were objective across studies and the comparison groups were relevant. However, interventions varied widely in regard to dose, length, and format, and the comparison groups differed across studies reducing quality indicating serious concerns for indirectness. | Serious concerns |
| Imprecision | A total of nine interventions were included with a median sample size of 205. The total sample of participants across all trials was 2,303. Six of nine trials significantly increased MVPA; however, this only included 44.5% of the total sample causing some borderline serious concerns with imprecision. | Serious concerns, borderline |
| Inconsistency | Six interventions demonstrated significant increases in moderate or MVPA within or between groups with some variability in mean/median differences. However, there are benefits from any amount of additional physical activity which demonstrates the clinical significance or these MVPA improvements. Further, the increase in physical activity from interventions outweigh any potential harm. No concerns for inconsistency were identified. | No concerns |
| Publication bias | A comprehensive search was conducted without any industry influence. The search did not include gray literature or studies not published in English. However, both significant and not significant studies were identified and included, reducing the likelihood that studies without significant findings were not published. Therefore, we judged the evidence as borderline no serious publication bias. | No concerns, borderline |
| ***Note.*** RCT = randomized controlled trials; RoB = risk of bias; MVPA = moderate to vigorous physical activity. | | |
